# Anlotinib Plus Osimertinib in Osimertinib‐Resistant Nonsquamous Nonsmall Cell Lung Cancer With Gradual Progression: A Retrospective Study

**DOI:** 10.1111/1759-7714.70071

**Published:** 2025-05-21

**Authors:** Yu Hua, Minghui Liu, Boshi Li, Hongbing Zhang, Zihe Zhang, Yanan Wang, Jinghao Liu, Xin Li, Yongwen Li, Sen Wei, Hongyu Liu, Jun Chen

**Affiliations:** ^1^ Department of Lung Cancer Surgery Tianjin Medical University General Hospital Tianjin China; ^2^ Tianjin Key Laboratory of Lung Cancer Metastasis and Tumor Microenvironment Tianjin Lung Cancer Institute, Tianjin Medical University General Hospital Tianjin China

**Keywords:** anlotinib, nonsquamous nonsmall cell lung cancer, osimertinib, resistance

## Abstract

**Background:**

Previous studies have shown that anlotinib plus third‐generation epidermal growth factor receptor tyrosine kinase inhibitors (EGFR‐TKIs) overcome acquired resistance to EGFR‐TKIs in patients with advanced EGFR‐mutant nonsmall cell lung cancer (NSCLC). This study aimed to retrospectively evaluate whether anlotinib plus osimertinib overcame acquired resistance in patients with nsq‐NSCLC who gradually progressed after first‐line EGFR‐TKI treatment.

**Methods:**

This study included patients with nsq‐NSCLC who developed gradual progression after first‐line osimertinib treatment, underwent an anlotinib plus osimertinib regimen in Tianjin Medical University General Hospital, and had available data from October 8, 2020 to October 14, 2023. Outcomes included the efficacy, assessed by progression‐free survival (PFS), of anlotinib plus osimertinib treatment (PFS1) and prior osimertinib treatment (PFS2), to disease progression, objective response rate (ORR), disease control rate (DCR), and safety as assessed by the incidence of treatment‐related toxicities.

**Results:**

A total of 28 patients with nsq‐NSCLC were included, with a median follow‐up of 12 months (range, 7.8–16.2). Treatment with anlotinib plus osimertinib led to a median PFS1 of 10.0 months (95% confidence interval [CI], 8.4–11.6). With a median follow‐up from prior osimertinib therapy of 31.5 months (range, 20.8–42.2), the median PFS2 was 22.0 months (95% CI, 17.5–26.5). The ORR to combination therapy was 3.6% (95% CI, 0.2–20.2) and the DCR was 85.7% (95% CI, 67.3–96.0). All patients experienced treatment‐related toxicities, with 10.7% showing grade 3, and none were grade ≥ 4.

**Conclusions:**

Anlotinib plus osimertinib exhibited encouraginsg anti‐tumor activity and had a manageable safety profile in patients with nsq‐NSCLC showing gradual progression on osimertinib.

## Graphical abstract

Previous studies have shown that anlotinib plus third‐generation epidermal growth factor receptor tyrosine kinase inhibitors (EGFR‐TKIs) overcome acquired resistance to EGFR‐TKIs in patients with advanced EGFR‐mutant nonsmall cell lung cancer (NSCLC). This study included patients with nsq‐NSCLC who developed gradual progression after first‐line osimertinib treatment and underwent anlotinib plus osimertinib. A total of 28 patients with nsq‐NSCLC were included, with a median follow‐up of 12 months (range, 7.8–16.2). Treatment with anlotinib plus osimertinib led to a median PFS1 of 10.0 months. With a median follow‐up from prior osimertinib therapy of 31.5 months, the median PFS2 was 22.0 months. The ORR to combination therapy was 3.6% and the DCR was 85.7%. All patients experienced treatment‐related toxicities, with 10.7% showing grade 3 and none were grade ≥ 4. Anlotinib plus osimertinib exhibited encouraging anti‐tumor activity and had a manageable safety profile in patients with nsq‐NSCLC showing gradual progression on osimertinib.

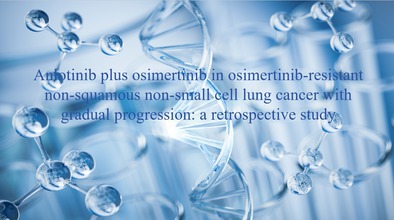

## 1 Introduction

Nonsmall cell lung cancer (NSCLC) is the most prevalent histological type of lung cancer, representing over 80% of all cases [1, 2] of which more than half are classified as nonsquamous (nsq)‐NSCLC [3, 4]. Most patients are diagnosed at an advanced stage and have a poor prognosis. Epidermal growth factor receptor tyrosine kinase inhibitors (EGFR‐TKIs) have become the first‐line treatment of choice for patients with NSCLC harboring EGFR‐sensitizing mutations. While EGFR‐TKIs initially improved the prognosis and quality of life of patients, overall cure and survival rates remain low due to inevitably acquired resistance [5, 6]. Patients tend to experience disease progression around 9–13 months after receiving treatment with first‐generation EGFR‐TKIs [7].

Osimertinib is an irreversible third‐generation EGFR‐TKI that can overcome EGFR T790M mutation–mediated resistance. It has shown better efficacy and safety than first‐generation EGFR‐TKIs for patients with EGFR‐mutant NSCLC in a first‐line setting [8]. Therefore, osimertinib has become the main treatment for EGFR‐mutated NSCLC, in both first‐ and second‐line settings [9, 10]. However, previous studies have indicated that subsequent clinical progression after treatment with osimertinib is inevitable and remains a major therapeutic challenge [11, 12, 13, 14]. The majority of patients choose to receive traditional chemotherapy as the main subsequent treatment after progression. However, only limited benefits are obtained, with a post‐progression survival of 7.8 months [15], leaving a huge demand for novel strategies.

Anti‐angiogenic drugs stop or slow NSCLC progression by targeting angiogenic signaling pathways and blocking the formation of new blood vessels necessary for tumor growth [16]. To put off or overcome any acquired resistance to osimertinib, combinations of anti‐angiogenic drugs with osimertinib have been tried in clinical trials. However, prospective studies have unfortunately failed to show an obvious survival benefit in using osimertinib plus several anti‐angiogenic drugs (bevacizumab, ramucirumab) [17, 18, 19]. Due to the heterogeneity of lung cancer and according to the nature of progression after EGFR‐TKI resistance, it is divided into three clinical modes: dramatic, gradual, and local progressions [20]. Of these, the gradual progression group showed a relatively better clinical benefit [21]. Therefore, researchers have begun to further explore the efficacy of EGFR‐TKI plus anti‐angiogenic drug regimens in this subgroup in order to provide a more effective and safer treatment option for this population [22, 23]. Most studies of such combination therapies for this special group of patients have been retrospective, and high‐level evidence published to prove efficacy is still lacking. Anlotinib, a multitargeted small‐molecule TKI, effectively combats tumor angiogenesis and inhibits tumor growth by inhibiting vascular endothelial growth factor receptor, platelet‐derived growth factor receptor, fibroblast growth factor receptor, and c‐Kit signaling pathways. Based on its anti‐angiogenic activity and anti‐tumor efficacy, anlotinib has been approved for the treatment of advanced NSCLC [24]. In addition, prior studies have shown that anlotinib plus other EGFR‐TKIs (gefitinib, afatinib) overcome acquired resistance to EGFR‐TKI in patients with advanced EGFR‐mutant NSCLC [25, 26]. However, evidence is lacking on the effectiveness of combining anlotinib with osimertinib in patients experiencing gradual progression following initial osimertinib treatment.

Therefore, in this retrospective study, we comprehensively report on the efficacy and safety of combining anlotinib with osimertinib in patients with nsq‐NSCLC who developed resistance and progressed gradually after initial treatment with osimertinib. We present this article in accordance with the STROBE reporting checklist.

## 2 Methods

### 2.1 Study Design and Patients

This observational, retrospective study was conducted in the Department of Lung Cancer Surgery, Tianjin Medical University General Hospital (Tianjin, China). Patients with nsq‐NSCLC who showed gradual progression after osimertinib and who received anlotinib plus osimertinib from October 8, 2020 to October 14, 2023, were consecutively included. The study was approved by the Ethics Committee of Tianjin Medical University General Hospital (approval number: IRB2023‐YX‐324‐01). It was conducted in compliance with the 1964 Declaration of Helsinki and applicable local laws and regulations.

Patient inclusion criteria were: (1) aged ≥ 18 years; (2) had histologically or cytologically confirmed unresectable, stage III or IV nsq‐NSCLC; (3) had at least one radiologically measurable lesion according to Response Evaluation Criteria in Solid Tumors version 1.1 (RECIST v1.1); (4) had an Eastern Cooperative Oncology Group Performance Status (ECOG PS) of ≤ 2; (5) had an activated EGFR mutation (L858R, T790M or 19DEL); (6) had a life expectancy of ≥ 3 months; and (7) showed gradual progression after first‐line treatment with osimertinib. Gradual progression was defined as disease control ≥ 6 months, no significant increment of tumor burden, progressive involvement of nontarget lesions with a score ≤ 2 compared with the previous evaluation, and a symptom score ≤ 1 [19].

Exclusion criteria included: (1) had small cell lung cancer (including mixed small cell and NSCLC) or lung squamous cell carcinoma; (2) had an ECOG PS of > 2; (3) had contraindications to Anlotini, including uncontrolled hypertension, recent myocardial infarction, congestive heart failure, a high risk of active bleeding (e.g., gastrointestinal hemorrhage, coagulopathy), uncontrolled central nervous system metastases, or severe hypersensitivity reactions to anlotinib and its excipients; and (4) had dramatic progression after first‐line treatment with osimertinib. Dramatic progression was defined as disease control ≥ 3 months, rapid progression of multiple target lesions or progressive involvement of nontarget lesions with a score > 2 compared with a previous evaluation, and a symptom score > 1 [19].

### 2.2 Therapy and Data Collection

All patients had received 8 mg anlotinib plus 80 mg osimertinib orally once daily in a 3‐week treatment cycle, with 2 consecutive weeks of treatment and 1 week of rest, until disease progression. Dose adjustment was performed according to drug instructions. When grade 3 hematologic toxicity or grade 2 or higher nonhematologic toxicity occurred, treatment was temporarily suspended and symptomatic treatment was given at the discretion of the physician until toxicity decreased to ≤ grade 1.

Patients' demographic and clinical characteristics (e.g., age, gender, smoking status, original EGFR mutation types, clinical stage, carcinoembryonic antigen [CEA] levels, metastasis, tumor diameter, and ECOG PS), tumor response data, survival status, and safety were collected retrospectively. All data were obtained from electronic medical records and follow‐up visits made by telephone. Patients' records were manually reviewed to confirm drug administration and the documentation of adverse events. Data were then pooled into a single secure database.

### 2.3 Outcomes

Efficacy outcomes were: progression‐free survival (PFS) of combination therapy (PFS1; defined as the time from starting anlotinib plus osimertinib treatment to the date of disease progression on combination therapy from any cause); PFS2 (defined as the time from prior osimertinib to the date of disease progression on combination therapy from any cause); and objective response rate (ORR) and disease control rate (DCR) after combination therapy per RECIST v1.1. The ORR was defined as the proportion of patients showing a complete response (CR) or partial response (PR) in two consecutive assessments collected at least 4 weeks apart. The DCR was defined as the proportion of patients showing a CR, PR, or stable disease (SD). Tumor response was assessed by physicians using low‐dose spiral computed tomography according to RECIST v1.1. Safety was assessed by treatment‐related toxicities recorded during combination therapy. Toxicity was graded using the National Cancer Institute Common Terminology Criteria for Adverse Events version 5.0 (CTCAE v5.0).

### 2.4 Statistical Analysis

Statistical analysis was performed using SPSS 25.0 (IBM Corporation, Armonk, NY, USA) and GraphPad Prism 9 (GraphPad Software, La Jolla, CA, USA). Demographic and clinical characteristics were summarized descriptively as a median (range) for continuous variables and as a number (%) for categorical variables. Survival data were estimated using the Kaplan–Meier method with corresponding two‐sided 95% confidence intervals (CIs). For survival analysis, patients without disease progression or death were censored at the date of the last follow‐up. A log‐rank test was applied to compare PFS between subgroups. Hazard ratios and corresponding 95% CIs were estimated from a Cox model. Response data (ORR and DCR) were calculated using a Clopper–Pearson method and compared with Fisher's precision probability test for subgroup analysis. Corresponding 95% CIs were calculated using a normal approximation to the binomial distribution. A two‐tailed test with a significance level of *p* < 0.05 was considered statistically significant (unless otherwise stated).

## 3 Results

### 3.1 Patient Characteristics

**FIGURE 1:**
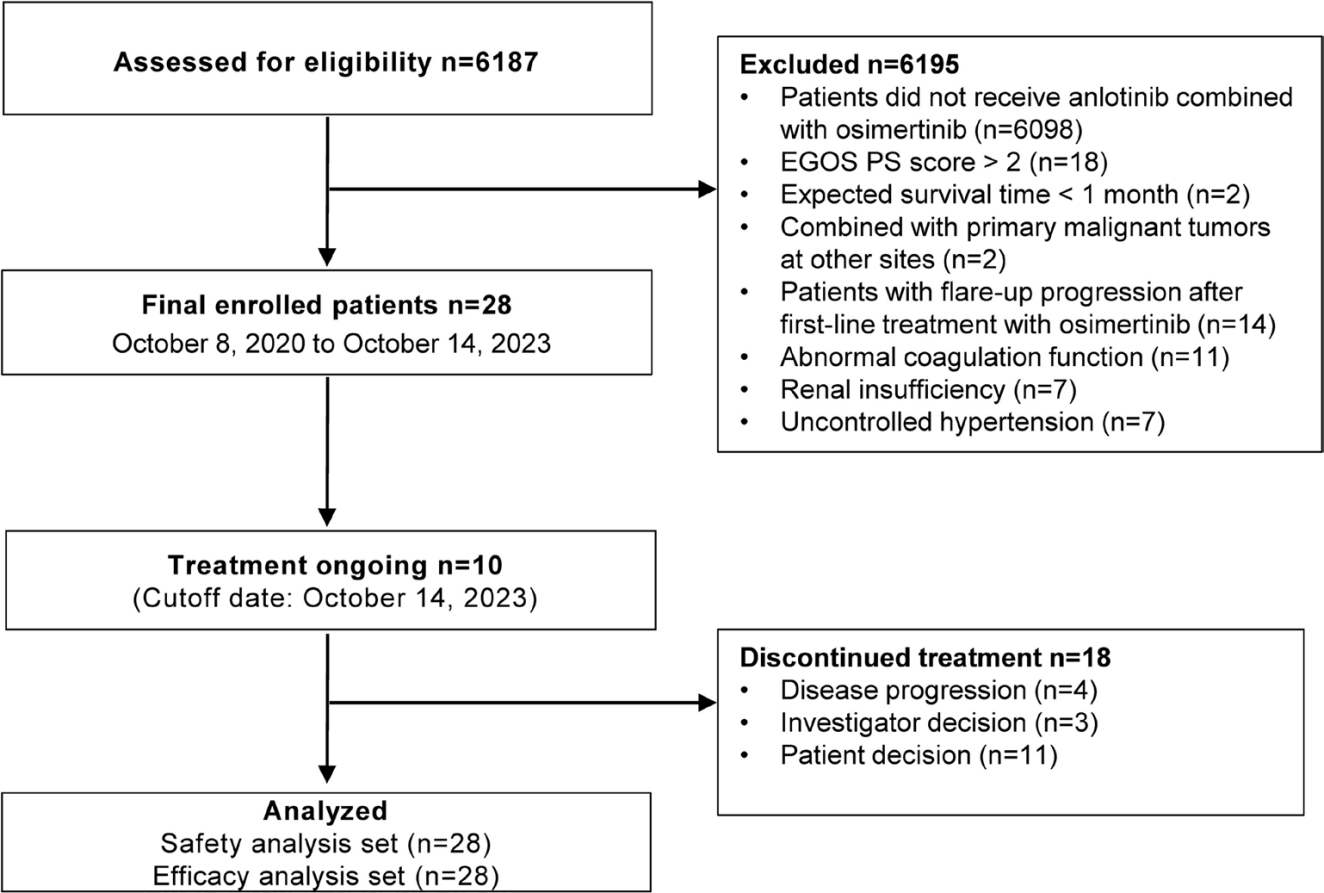
Flow of patients.

Between October 8, 2020 and October 14, 2023, a total of 28 patients (12 males) were included in this study (Figure 1), and all patients underwent tumor biopsy as part of their clinical evaluation. The 28 patients had a median age of 67 years (range, 40–80 years; Table 1). All patients (100%) had a pathological type of lung adenocarcinoma. Most patients were nonsmokers (60.7%) and were clinical stage IV (53.6%) and ECOG PS ≤ 1 (75.0%). Regarding EGFR mutation type, 14 (50%) patients had an exon 21 L858R mutation, nine (32.1%) had a T790M mutation, and five (17.9%) had an exon 19 deletion (19Del). Additionally, the co‐existence of EGFR and other mutations was detected in 13 (46.4%) patients. Brain metastases occurred in nine (32.1%) patients.

**TABLE 1:** Patient characteristics.

Characteristics	Patients (*n* = 28)
Age, years	
Median	67 (40–80)
< 60	4 (14.3)
≥ 60	24 (85.7)
Pathological type	
Lung adenocarcinoma	28 (100)
Sex	
Male	12 (42.9)
Female	16 (57.1)
ECOG PS	
0–1	21 (75.0)
2	7 (25.0)
Smoking status	
Smoker	11 (39.3)
Nonsmoker	17 (60.7)
Original EGFR mutation types	
L858R	14 (50.0)
T790M	9 (32.1)
19Del	5 (17.9)
Concomitant mutations	
TP53	5 (17.9)
Other mutations	8 (28.6)
Clinical stage	
III	13 (46.4)
IV	15 (53.6)
Brain metastasis	
Yes	9 (32.1)
No	19 (67.9)
CEA	
< 5	2 (7.1)
≥ 5	26 (92.9)
Tumor diameter	
≤ 3	11 (39.3)
3–5	14 (50.0)
> 5	3 (10.7)

*Note:* Data are median (range) and *n* (%).

Abbreviations: CEA, carcinoembryonic antigen; ECOG PS, Eastern Cooperative Oncology Group performance status; EGFR, epidermal growth factor receptor.

### 3.2 Tumor Response

**FIGURE 2:**
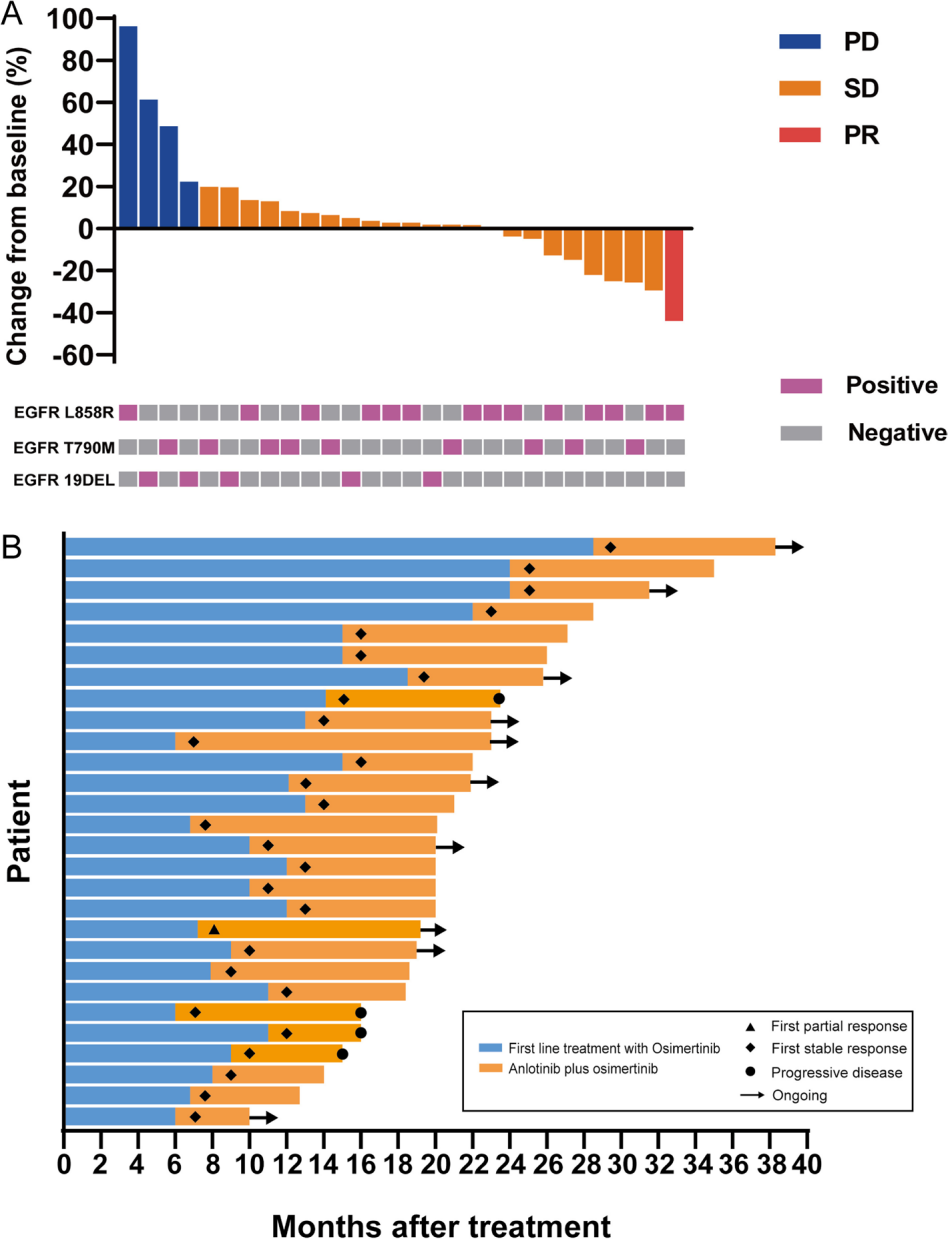
Tumor responses. (A) Maximum change from baseline in the sum of diameters of target lesions. (B) Swimmer plot of time on treatment.

The post‐treatment response was evaluated for all 28 included patients, being evaluable for efficacy analysis. One (3.6%) patient achieved a PR after combination therapy (Table 2), resulting in an ORR of 3.6% (95% CI, 0.2–20.2). An additional 23 (82.1%) patients showed SD as the best response. Thus, the DCR was 85.7% (95% CI, 67.3–96.0; Figure 2A and Table 2). The time period of treatment for all patients is shown in Figure 2B. As for the data cut‐off, out of the 28 patients who received anlotinib plus osimertinib treatment, 24 (85.7%) patients had their tumor progression controlled, four (14.3%) patients discontinued combination treatment due to disease progression, and 14 (50%) patients discontinued combination therapy for various reasons. Durable responses for over 6 months were observed in 22 (78.6%) patients and responses were ongoing in 10 (35.7%) patients. The longest duration of response was 17 months and was observed in one patient with an EGFR T790M/ERBB2 mutation.

**TABLE 2:** Clinical response to anlotinib plus osimertinib in nsq‐NSCLC patients with gradual progression on osimertinib.

Efficacy evaluation	Patients (*n* = 28)
Best response, *n* (%)	
PR	1 (3.6)
SD	23 (82.1)
PD	4 (14.3)
ORR, % (95% CI)	3.6 (0.2–20.2)
DCR, % (95% CI)	85.7 (67.3–96.0)

*Note:* Data are *n* (%), % (95% CI), or median (95% CI).

In a subgroup analysis of tumor response, no significant association was found between DCR and baseline characteristics, including age, gender, EGFR mutation types, smoking status, concomitant mutations, brain organ metastasis, CEA levels, and tumor diameter (Figure S1). Stage IV patients demonstrated a significantly higher DCR in comparison to stage III patients (*p* = 0.035). Notably, patients who were aged < 60 years, with clinical stage IV, co‐occurring EGFR and TP53 mutations, brain metastases, ECOG PS ≤ 1, baseline CEA < 5 μg/L, and a tumor diameter > 5 mm achieved DCRs of up to 100%. Nevertheless, the significance regarding subgroup results was preliminary due to the limited number of patients and the retrospective nature of this study.

### 3.3 Survival Outcomes

**FIGURE 3:**
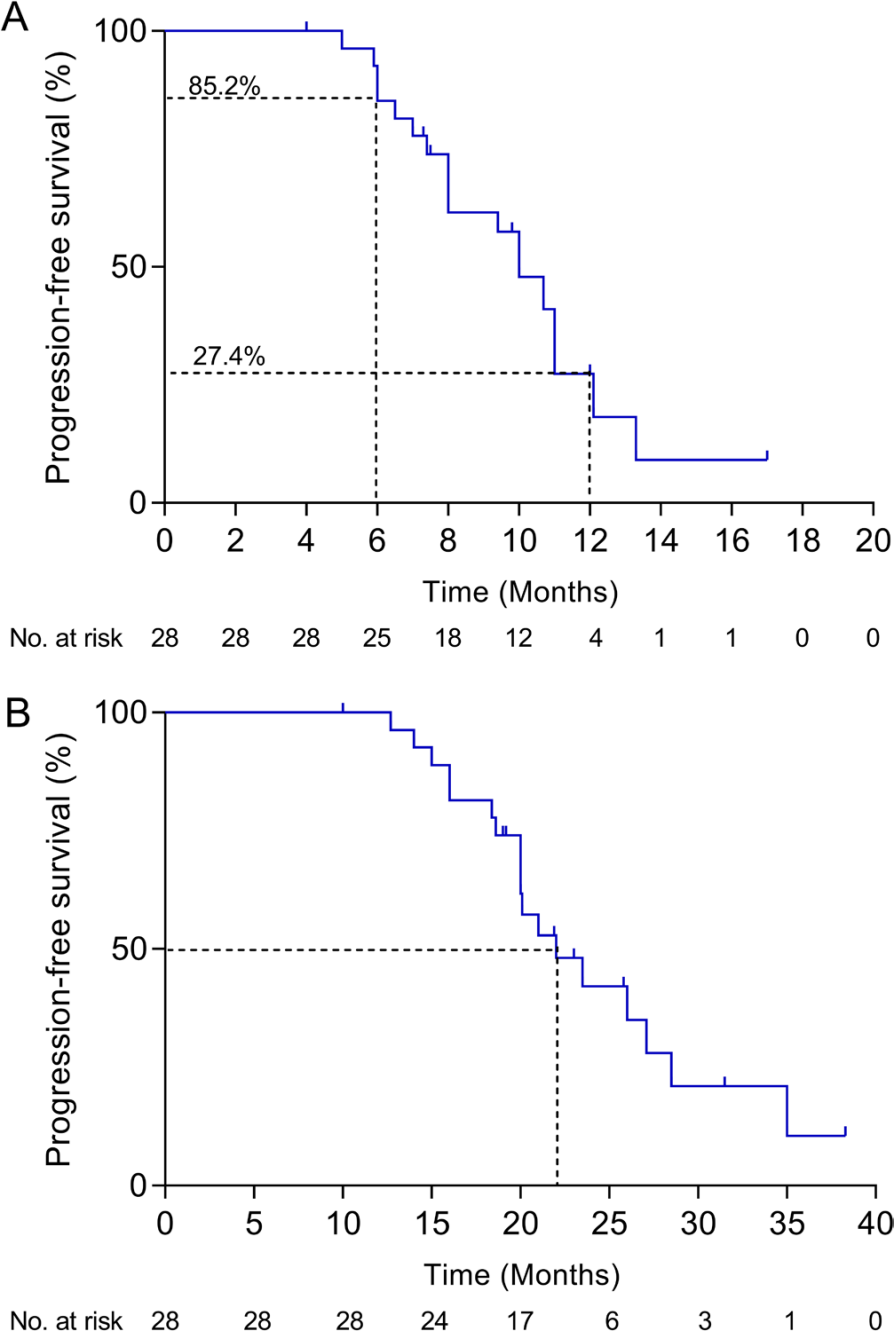
Kaplan–Meier survival curves of progression‐free survival. (A) Progression‐free survival after anlotinib plus osimertinib. (B) Progression‐free survival from first‐line osimertinib.

The data cutoff date for survival was October 14, 2023. The median follow‐up of the 28 included patients from the start of anlotinib plus osimertinib was 12.0 months (range, 7.8–16.2). A total of four (14.3%) patients with gradual progression showed dramatic progression after combination therapy; thus, a combination of anlotinib and osimertinib led to a median PFS1 of 10.0 months (95% CI, 8.4–11.6 months), with an estimated 6‐month PFS rate of 85.2% (95% CI, 65.2–94.2; Figure 3A). From initial osimertinib treatment, the 28 patients had a median follow‐up of 31.5 months (range, 20.8–42.2). The median PFS2 from first‐line osimertinib to progression with combination therapy was 22.0 months (95% CI, 17.5–26.5 months; Figure 3B). With a relatively short follow‐up, the median OS has yet to be immature.

After stratifying according to baseline characteristics, the median PFS1 was significantly longer in nonsmokers (11.0 months [95% CI, 10.0–12.0 months]; Figure S2) than in smokers (8.0 months [95% CI, 5.3–10.7 months], *p* = 0.048). Other analyzed clinical factors showed no significant association with median PFS1 (*p* > 0.05; Figure S2). For instance, patients with an EGFR L858R mutation (11.0 months; 95% CI, 7.9–14.2) had a numerically longer PFS than patients with an EGFR T790M mutation (10.0 months; 95% CI, 6.5–13.5) or an EGFR 19del mutation (9.4 months; 95% CI, 5.1–13.7), although this was not statistically different (*p* = 0.569).

To further identify patients who may benefit more from this combination, patients were categorized into two subgroups according to co‐occurring mutations: co‐occurring EGFR and TP53 mutations (*n* = 5) vs. co‐occurring EGFR and other mutations (*n* = 8). The mutation type was not associated with either PFS1 (11.0 months vs. 10 months; *p* = 0.725; Figure S3A) or PFS2 (31.75 months vs. 19.2 months; *p* = 0.060; Figure S3B) with our combination therapy.

### 3.4 Safety

During combination therapy, all patients (100%) experienced treatment‐related toxicities. The most commonly observed toxic reactions were diarrhea (60.7%), poor appetite (50.0%), nausea (46.4%), hypertension (35.7%), and vomiting (32.1%). Grade 3 toxicities occurred in three patients (10.7%), including diarrhea, nausea, and vomiting (3.6% each; Table 3). No patients experienced grade ≥ 4 toxicities or discontinued treatment due to treatment‐related toxicities. No dose adjustments were made for any of the patients throughout the treatment course. No treatment‐related deaths occurred after combination therapy.

**TABLE 3:** Incidence of TRAEs after anlotinib plus osimertinib therapy in nsq‐NSCLC patients with gradual progression on osimertinib.

Adverse event	Overall (*n* = 28), *n* (%)		
	All grades	Grade 1–2	Grade 3
Any events	28 (100)	25 (89.3)	3 (10.7)
Diarrhea	17 (60.7)	16 (57.1)	1 (3.6)
Poor appetite	14 (50.0)	14 (50.0)	0
Nausea	13 (46.4)	12 (42.8)	1 (3.6)
Hypertension	10 (35.7)	10 (35.7)	0
Vomiting	9 (32.1)	8 (28.5)	1 (3.6)
ALT increased	8 (28.6)	8 (28.6)	0
Cough	8 (28.6)	8 (28.6)	0
Constipation	8 (28.6)	8 (28.6)	0
Alopecia	7 (25.0)	7 (25.0)	0
AST increased	7 (25.0)	7 (25.0)	0
Weight decreased	6 (21.4)	6 (21.4)	0
Hyponatremia	5 (17.9)	5 (17.9)	0
Proteinuria	4 (14.3)	4 (14.3)	0
Hypokalemia	4 (14.3)	4 (14.3)	0

*Note:* Data are *n* (%). There were no grade 4–5 events.

Abbreviations: ALT, alanine aminotransferase; AST, aspartate aminotransferase.

## 4 Discussion

This study provided evidence of anti‐angiogenic drugs plus EGFR‐TKIs in nsq‐NSCLC with gradual progression when on third‐generation EGFR‐TKI osimertinib. The combination of anlotinib plus osimertinib has shown promising efficacy with a median PFS of 10.0 months and DCR of 85.7%. Even in patients with brain metastases, the regimen showed an effective tumor response with a DCR of 100%. In addition, the regimen demonstrated a tolerable safety profile with a low incidence of grade ≥ 3 adverse events (AEs). The results of this study support further validation of this regimen in clinical studies.

For those patients with nonsquamous nonsmall‐cell lung cancer (nsq‐NSCLC) who have disease progression after osimertinib treatment, there are a variety of treatment options currently available. Multiple treatment options exist and are approved including platinum‐pemetrexed; carboplatin/paclitaxel/atezolizumab/bevacizumab; carboplatin/pemetrexed+Amivantamab and Sintilimab+Bevacizumab+Chemo [27, 28]. Among the numerous treatment options after the progression of osimertinib treatment, the combination of osimertinib and anlotinib demonstrates certain advantages. The present study observed that a combination of anlotinib and osimertinib following osimertinib failure yielded an encouraging response (ORR, 3.6%; DCR, 85.7%) and promising survival signal (median PFS1, 10.0 months) in patients with nsq‐NSCLC showing gradual progression when on osimertinib. The survival benefit of the combination regimen in this study was superior to that previously reported with the addition of apatinib (median PFS, 8.2 months) [29] or savolitinib (median PFS, 7.6 months) [30] after gradual progression with first‐line EGFR‐TKIs. Notably, prior studies have reported that switching to combination therapy after progression is more effective than continuing EGFR‐TKI monotherapy [9]. Encouragingly, our regimen was more effective than previous regimens starting with prior therapy (median PFS, 22.0 vs. 20.9 months). The advantage here could potentially be due to the potent synergy between anlotinib and osimertinib, which reverses acquired resistance to osimertinib by targeting the c‐MET/MYC/AXL axis and downstream MAPK and PI3K/AKT signaling pathways [31]. However, although results appear promising, comparisons across studies should be interpreted with caution as study designs and sample sizes may differ.

Moreover, several meaningful findings were observed from subgroup analysis. We observed that a combination regimen showed superior anti‐tumor activity with a DCR of 100% in nsq‐NSCLC patients with brain metastases in this study, which may be attributed to the active effect of anlotinib in the brain. Anlotinib improved intracranial efficacy in patients with brain metastases and played a potential role in tumor control at intracranial sites [32]. Additionally, a subgroup analysis also demonstrated that our combination is more effective for nonsmokers than for former smokers, similar to that found with anlotinib plus pemetrexed in EGFR/ALK wild‐type advanced nsq‐NSCLC [33]. Compared with smokers, nonsmokers have a different genomic makeup and prevalent somatic mutations in EGFR, ERBB2, ALK, and ROS1, which may also contribute to their greater benefit from targeted therapy [34]. This study also found that stage IV patients benefited more from combination therapy compared to stage III patients.

Identifying populations that are more likely to benefit from biomarkers has great clinical importance. Although no association was found between EGFR L858R, T790M, and 19del and responses, an association between TP53 mutations and PFS was determined. Treatment with EGFR‐TKI showed worse efficacy in patients with TP53 mutations, as reported in previous studies [35, 36]. However, our combination regimen showed similar efficacy in both groups of patients with TP53 and other mutations. Although a statistical difference did not exist, our study revealed that patients with TP53 mutations who demonstrated slow progression on osimertinib and subsequently received combined treatment with osimertinib and anlotinib exhibited a longer PFS compared to patients with other mutations. Patients with TP53 mutations may potentially benefit from combination therapy. However, the sample size of this study is small and needs further verification.

A combination of anlotinib and osimertinib showed acceptable tolerability in this population. The reported treatment‐related toxicities were known and common, such as diarrhea, poor appetite, nausea, and hypertension, and were similar to monotherapy regimens with osimertinib or anlotinib [37, 38, 39], implicating that combination therapy in the present study did not increase risk. Based on the results of the ORCHARD study, in patients who showed an initial resistance to osimertinib and were subsequently put on additional necitumumab combination therapy, up to 44% experienced one or more grade ≥ 3 AEs, 31% experienced serious AEs, and 13% died [40]. By contrast, only 10.7% of grade 3 AEs were observed in the present study, suggesting that our combination regimen may be safer and acceptable. However, our study was retrospective, which would lead to an underestimation of the incidence of AEs. Additionally, the toxicities of the combination regimen were generally controllable and tolerable since these resolved soon after symptomatic treatment; no one discontinued study treatment because of toxicities. Overall, these findings suggest that combining anlotinib with osimertinib may be a safer treatment option in this population.

Although important discoveries were revealed by our study, several limitations still exist. First, the relatively small sample size of patients used in this study may lead to a bias in our results. Second, our study had its inherent limitations of having a retrospective and nonrandomized design. Finally, the limited observation period led to a lack of long‐term efficacy. Thus, further prospective trials are still warranted to validate our findings.

## 5 Conclusions

In conclusion, anlotinib plus osimertinib showed encouraging anti‐tumor activity and had a manageable safety profile in nsq‐NSCLC with gradual progression on osimertinib, providing a feasible and well‐tolerated treatment option for this population. Moreover, the finding may be considered evidence to further understand the application of anlotinib plus osimertinib as treatment for such patients. Further evaluation of this combination regimen needs to be made in larger randomized clinical trials in the future.

## Author Contributions

**Yu Hua:** software, validation, writing – review and editing. **Minghui Liu:** software, validation, writing – review and editing. **Boshi Li:** software, validation, writing – review and editing. **Hongbing Zhang:** data curation, methodology. **Zihe Zhang:** data curation, methodology. **Yanan Wang:** formal analysis, validation. **Jinghao Liu:** data curation, formal analysis. **Xin Li:** data curation, formal analysis. **Yongwen Li:** conceptualization, data curation, formal analysis, funding acquisition, investigation, project administration, resources. **Sen Wei:** investigation, methodology, project administration, resources, supervision, validation, writing – review and editing. **Hongyu Liu:** funding acquisition, investigation, methodology, project administration, resources, supervision, validation, writing – review and editing. **Jun Chen:** funding acquisition, investigation, methodology, project administration, resources, supervision, validation, writing – review and editing.

## Ethics Statement

The authors are accountable for all aspects of the work in ensuring that questions related to the accuracy or integrity of any part of the work are appropriately investigated and resolved. The study was approved by the Ethics Committee of Tianjin Medical University General Hospital (approval number: IRB2023‐YX‐324‐01). It was conducted in compliance with the 1964 Declaration of Helsinki and applicable local laws and regulations. As our study was noninterventional and retrospective, informed consent was waived by the committee or the institutional.

## Consent

The authors have nothing to report.

## Conflicts of Interest

The authors declare no conflicts of interest.

## Supporting information


**Figure S1.** Univariate analysis of factors influencing disease control rate.

**Figure S2.** Univariate analysis of factors influencing progression‐free survival.

**Figure S3.** Kaplan–Meier survival curves of progression‐free survival with co‐occurring mutation. (A) Progression‐free survival after anlotinib plus osimertinib. (B) Progression‐free survival from first‐line osimertinib.

## Data Availability

Data will be available upon reasonable request to the corresponding author.
