# Supplementary figures and images for: Anlotinib Plus Osimertinib in Osimertinib‐Resistant Nonsquamous Nonsmall Cell Lung Cancer With Gradual Progression: A Retrospective Study
Source: Thorac Cancer. 2025 May 21;16(10):e70071. doi: 10.1111/1759-7714.70071 (PMC12093106; doi:10.1111/1759-7714.70071)

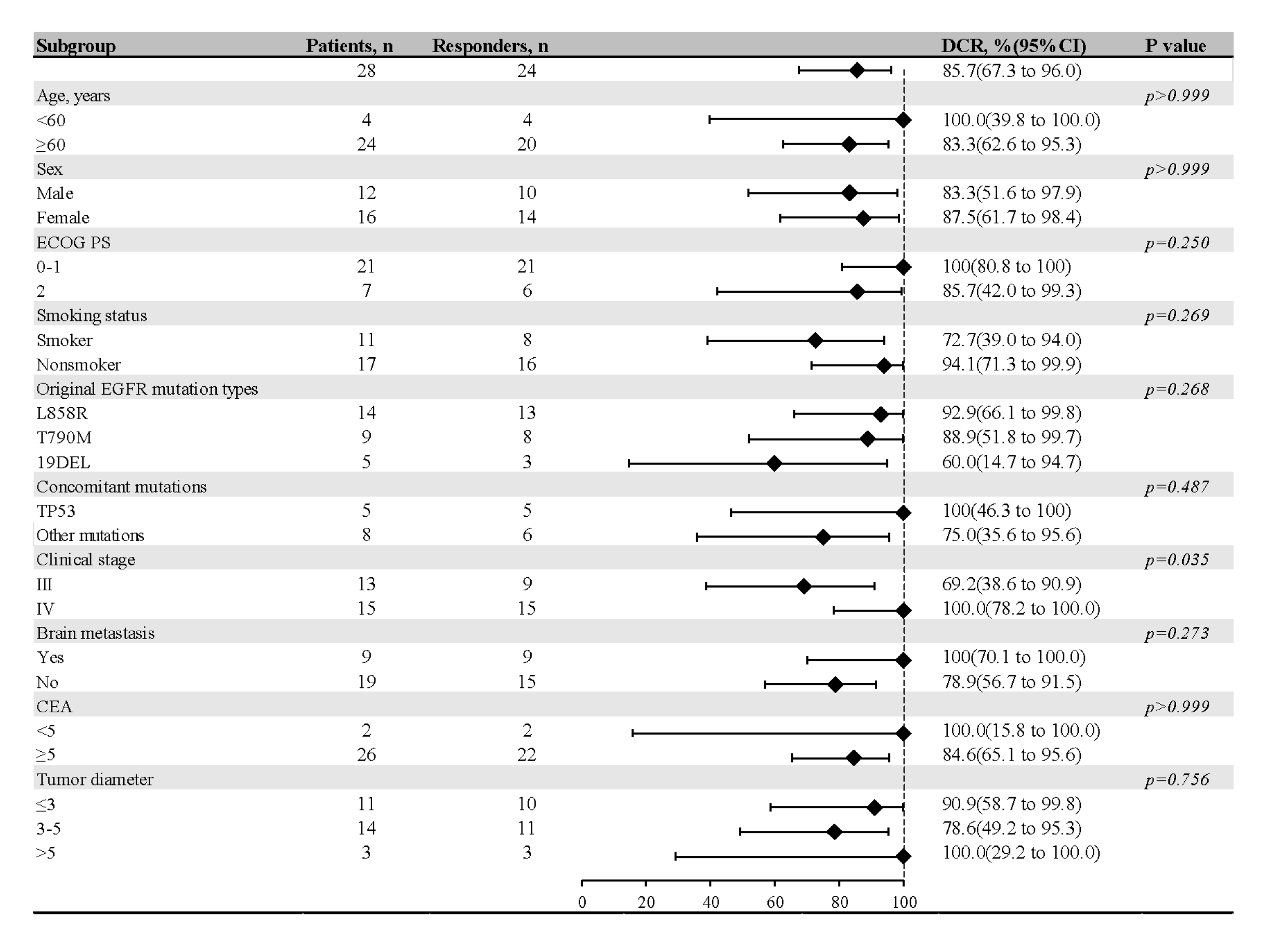

Supplement: Supplementary file 1 — Figure S1. Univariate analysis of factors influencing disease control rate. [file TCA-16-e70071-s001.tif]

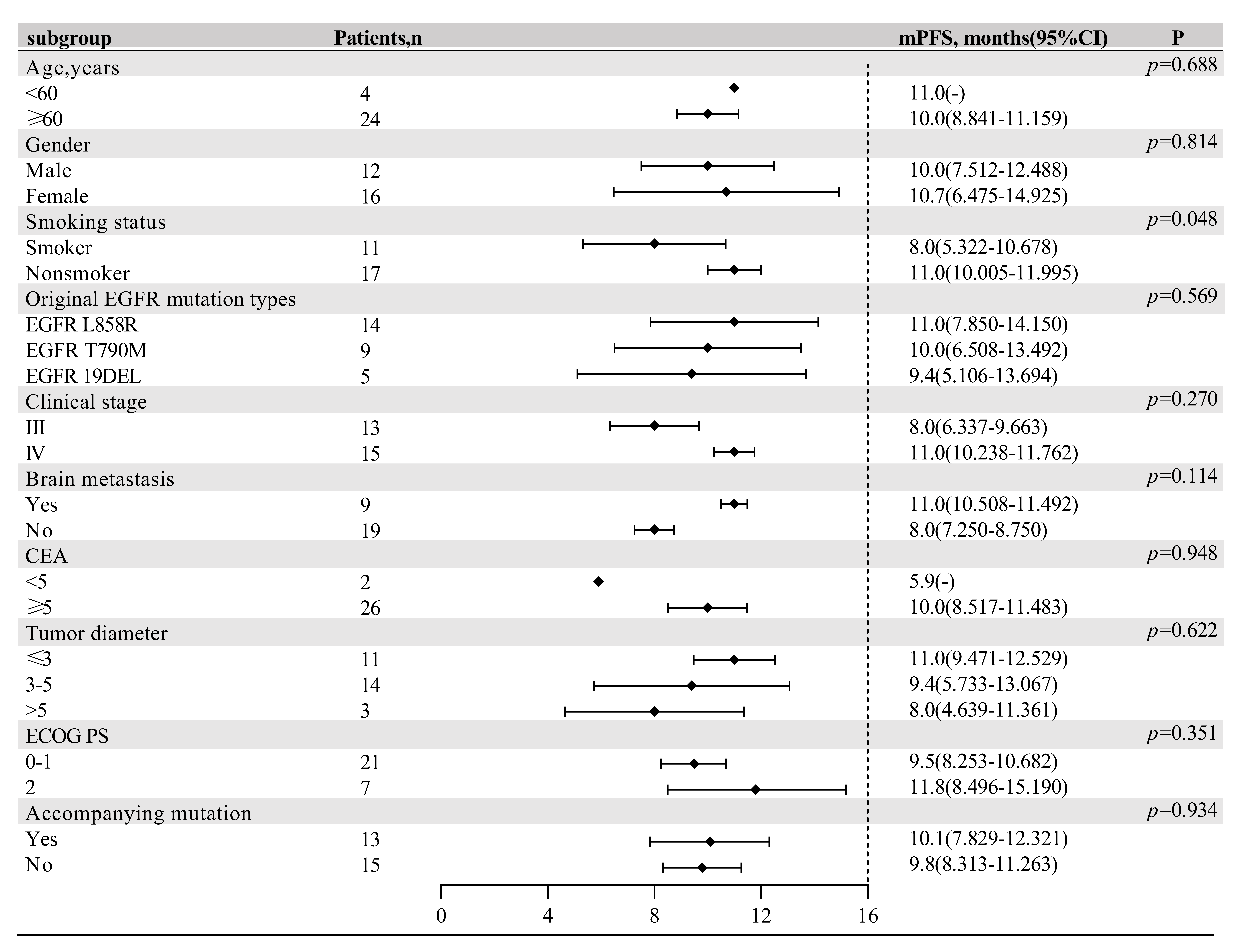

Supplement: Supplementary file 2 — Figure S2. Univariate analysis of factors influencing progression‐free survival. [file TCA-16-e70071-s003.tif]
